# Early nociceptive evoked potentials in symptomatic and asymptomatic transthyretin mutation carriers

**DOI:** 10.3389/fneur.2025.1677450

**Published:** 2025-11-17

**Authors:** Sara Massucco, Viola Bruzzone, Lucio Marinelli, Silvia Stara, Paola Mandich, Consuelo Venturi, Chiara Gemelli, Angelo Schenone, Massimo Leandri, Marina Grandis

**Affiliations:** 1 Department of Neuroscience, Rehabilitation, Ophthalmology, Genetics, Maternal and Child Health (DiNOGMI), University of Genoa, Genova, Italy; 2 IRCCS Ospedale Policlinico San Martino, Genova, Italy

**Keywords:** transthyretin amyloidosis, neuropathy, pain-related evoked potential, nociceptive evoked potential, skin biopsy, small fiber, electrochemical skin conductance

## Abstract

**Introduction:**

Small fiber involvement is an early feature of hereditary transthyretin amyloidosis with polyneuropathy (ATTRv-PN), but neurophysiological assessment remains challenging due to the lack of reliable methods. Nociceptive evoked potentials (NEPs), recently introduced to evaluate the nociceptive pathway, were employed here to identify potential neurophysiological abnormalities in ATTRv-PN.

**Methods:**

Fourteen transthyretin mutation carriers (eight ATTRv-PN, six asymptomatic) underwent a cross-sectional evaluation, including clinical scales, NEPs, pain-related evoked potentials (PREPs), somatosensory evoked potentials, electrochemical skin conductance, and skin biopsy. Evoked potentials were compared with 14 age-matched healthy controls.

**Results:**

NEPs were abnormal in 75% of ATTRv-PN patients and 50% of asymptomatic carriers, either absent or delayed. In contrast, PREPs were impaired in only 37.5% of ATTRv-PN patients and none of the asymptomatic carriers. All but two ATTRv-PN patients, and none of the asymptomatic carriers, showed reduced electrochemical skin conductance at the palms and soles.

**Discussion:**

NEPs appear valuable for assessing small fiber impairment in ATTRv-PN. They may enable early detection of disease onset in asymptomatic carriers and provide a means to monitor disease progression in ATTRv-PN.

## 1 Introduction

Hereditary transthyretin amyloidosis (ATTRv, v for variant) is the most common familial amyloidosis, affecting approximately 50,000 people worldwide (1). In this multisystemic disease, the extracellular deposition of amyloid fibrils in the peripheral nervous system results in progressive axonal degeneration (2). The typical length-dependent sensory-motor polyneuropathy is frequently preceded by small fiber involvement, affecting pain and temperature sensations as well as autonomic functions (3). Conventional nerve conduction studies are usually unremarkable in early stages as they assess large fibers only, resulting in unrecognized small fiber impairment (4) and diagnostic delays (5).

Intraepidermal nerve fiber density (IENFD) is reduced even in asymptomatic transthyretin (TTR) mutation carriers (6), highlighting the need for functional assessment of small Aδ and C fibers to enable early diagnosis and timely therapy. Unfortunately, available neurophysiological techniques are limited in number, reliability, and feasibility, making them unsuitable for routine clinical use. Recently, a micropatterned electrode with a 150 μm inter-rail gap (150 IDE; Italian Patent n.1425199, WO/2015/186087) has been developed to deliver electric stimuli to intraepidermal free nerve endings selectively (7). Selectivity was demonstrated through near-nerve recordings (8) and selective nerve blocks (9). This approach enables the excitation of Aδ fibers and potentially C fibers while avoiding the activation of deeper Aβ fibers (7). The 150 IDE allows recording of early Nociceptive Evoked Potentials (NEPs), with the N40 component reflecting the first arrival of the fast Aδ volley at the primary somatosensory cortex (7–9). NEPs offer three key advantages: (1) they do not require subject cooperation, (2) allow for high stimulation rates, up to 1 Hz for as many as 1,000 stimuli, and (3) are safe. These ensure high reliability, almost comparable to the somatosensory evoked potential (SEP) N20. NEP N40 represents the nociceptive counterpart of SEP N20, with the former reflecting the cortical arrival of fast nociceptive afferents and the latter corresponding to the fastest somatosensory volley (8). Other electrical stimulation methods, whether surface-based or minimally invasive (10–12), intended to activate free nerve endings, lack selectivity (7, 13), and only evoke long-latency cortical responses (pain-related evoked potentials, PREPs) (10). PREPs are endogenous, event-related responses, not directly linked to the physical properties of the stimulus, and originate from widespread cortical areas. Their clinical utility is limited by high variability in amplitude and latency and strong habituation effects that preclude repetitive stimulation and require randomized, time-spaced stimuli (8).

This study explored whether NEPs, given their reliability in assessing the nociceptive pathway from the periphery to the parietal cortex, could support early diagnosis of ATTRv with polyneuropathy (ATTRv-PN). NEPs were compared with other published methods for detecting small fiber impairment. The ultimate goal was to identify a reliable, clinically feasible biomarker for integration into neurophysiological practice.

## 2 Materials and methods

### 2.1 Study population

The study included 14 individuals with TTR pathogenic variants (eight with ATTRv-PN and six asymptomatic) and 14 age-matched healthy controls. Carriers were considered asymptomatic if they met the following criteria: (a) no sensory or autonomic symptoms (Lauria score < 2) (14), (b) normal neurological examination with a Neuropathy Impairment Score (NIS) of 0–1, and (c) normal nerve conduction studies. The predicted age of disease onset (PADO) was assessed based on the average age of onset in affected family members and the age of onset reported in the literature for each specific mutation (15). The time to PADO (delta-PADO) was calculated by subtracting the carrier’s age from the PADO, providing an estimate of the remaining years before overt disease.

Subjects with other causes or risk factors for peripheral neuropathy, such as diabetes, vitamin B9 or B12 deficiency, paraproteinemia, hypothyroidism, or alcohol consumption exceeding two drinks/day, were excluded.

The study was conducted in accordance with the Declaration of Helsinki and was approved by the Regional Ethics Committee of Liguria (Approval No. 14/2019; date of approval: 03 November 2020). All participants provided written informed consent.

### 2.2 Study design

The study was designed as a single-center, cross-sectional investigation. The following demographic and clinical features were collected: age, sex, TTR mutation, age at disease onset, disease duration or delta-PADO, and disease-modifying therapy. Participants underwent: (1) neurological examination with clinical scales and questionnaires; (2) autonomic assessment, including evaluation of orthostatic hypotension (brachial blood pressure measured at rest and 60 and 180 s after standing) and symptom assessment using the Compound Autonomic Dysfunction Test (CADT) and the Composite Autonomic Symptom Score-31 (COMPASS-31); (3) peripheral nerve conduction studies, including radial nerve sensory antidromic conduction; (4) NEP, PREP, and SEP recordings; (5) electrochemical skin conductance (ESC); and (6) skin biopsy.

### 2.3 Clinical scales and questionnaires

Neuropathy severity was assessed using NIS (0–244) and NIS-lower limb (NIS-LL; 0–88), where higher scores indicate greater impairment (16). Functional impact was measured with the Polyneuropathy Disability (PND) scale (17). Autonomic symptoms were evaluated using CADT (0–20 in males, 0–16 in females, with lower scores indicating more severe symptoms) and COMPASS-31 (0–100, with higher scores indicating more severe symptoms) (18, 19). To account for sex-based CADT differences, percent changes were used for combined analyses. Quality of life was assessed with the Norfolk-Diabetic Neuropathy Quality of Life Questionnaire (Norfolk-DN QoL; 0–156), with higher scores reflecting poorer quality of life (20). Neuropathic pain was defined as Douleur Neuropathique 4 (DN4) ≥ 4 (21).

### 2.4 NEPs

#### 2.4.1 Stimulation

The 150 IDE (15 × 15 mm flexible version, Bionen S.A.S., Florence, Italy) was applied to the hairy skin on the dorsum of the dominant hand, between the first and second metacarpal bones, after cleaning the skin with ethanol to remove moisture and lipid film. The electrode was secured with tape. Bursts of 10 0.2-ms pulses, with a period of 1 ms, were generated by a TG2511 arbitrary waveform generator (Thurlby Thandar Instruments Ltd., Huntingdon, UK) triggering a constant current stimulator (DS7A, Digitimer Ltd., UK). Intensity was set at 1.5 times the perception threshold, producing a local pinprick sensation. Sustained rhythmic stimulation was performed at a rate of 0.83 Hz, with 500–1,000 stimuli, in a temperature-controlled room. Participants were instructed to lie supine with their eyes closed and report any changes in stimulus perception. Due to the minute interrail gap of the 150 IDE, skin conditions could affect impedance, which was continuously monitored (optimal: 16–30 kΩ).

#### 2.4.2 Signal recording and handling

Responses were recorded from C3’/C4’-Fz using subdermal needle electrodes (Bionen S.A.S., Florence, Italy). Signals were amplified 100,000 times with a 0.1–2000 Hz bandpass (second-order Butterworth analog filter; LT amplifiers, Vertigo, Genova, Italy), then digitized (NI PCIe-6320, X Series Multifunction DAQ, 16-bit, 250 KS/s; National Instruments, Austin, TX, USA). The acquisition window was 800 ms (300 ms pre- and 500 ms post-stimulus) with a 25K/s sampling rate, using custom software developed in LabVIEW^®^ 2019 (National Instruments, Austin, TX, USA). Records with biological artifacts or electromagnetic interference were discarded during offline analysis.

### 2.5 PREPs

#### 2.5.1 Stimulation

Electrodes and sites were the same as in section 2.4.1. Stimuli matched NEP parameters but were delivered at random intervals (5–14 s) to prevent the habituation typical of PREPs. Only 30 responses were collected to minimize habituation. After each stimulus, subjects rated the perceived intensity on a scale from 0 (not perceived) to 10 (maximum imaginable; optimal: 2–3).

#### 2.5.2 Signal recording and handling

N1 responses were recorded from C3’/C4’-Fz, and N2-P2 from Cz-Au1/2, using subdermal needle electrodes. Amplification, storage, and analysis followed the same procedures as in section 2.4.2.

### 2.6 SEPs

#### 2.6.1 Stimulation

For radial SEPs, self-adhesive Ag/AgCl electrodes were placed on the hand dorsum, between the first and second metacarpals. The same burst of electrical pulses described in section 2.4.1 was delivered at 1.5 × sensory threshold with the same rate.

Median SEPs were obtained by stimulating the median nerve at the wrist at motor threshold with single 0.2-ms pulses using Ag/AgCl electrodes.

#### 2.6.2 Signal recording and handling

Responses were recorded from C3’/C4’-Fz using subdermal needle electrodes. Acquisition parameters matched those in section 2.4.2.

### 2.7 ESC measurement

The Sudoscan device (Impeto Medical) was used to measure electrochemical skin conductance (ESC) of the palms and soles, which results from chloride ion flow in response to a locally applied low-voltage direct current (<4 V) and is expressed in microsiemens (μS). Values ≥60 μS for the hands and ≥70 μS for the feet were considered normal (22).

### 2.8 Skin biopsy

Skin samples were collected with a 3-mm punch under sterile conditions after topical lidocaine anesthesia. Hairy skin biopsies were taken from the distal leg (~10 cm above the lateral malleolus) and processed following standardized protocols (23). Specimens were fixed overnight in 2% paraformaldehyde-lysine-periodate at 4 °C, cryoprotected, and cut into 50-μm free-floating sections. Sections were stained using a rabbit antibody against the panneuronal marker protein gene product 9.5, followed by biotinylated anti-rabbit IgG, avidin-biotin ABC complex, and peroxidase substrate, producing a blue-gray reaction. Intraepidermal nerve fibers were counted at 40× magnification under light microscopy on at least three sections per biopsy. Only fibers crossing the dermal-epidermal junction were counted; secondary branching and fragments were excluded. IENFD was expressed as fibers/mm, with values below the fifth percentile for age and sex considered abnormal (24). The pathologist (A.S.) was blinded to the diagnosis.

### 2.9 Data analysis

To minimize selection bias, all consecutive eligible patients were enrolled. Continuous variables are reported as medians (ranges or interquartile ranges) or means (standard deviations), and categorical variables as counts. Normality was assessed with the Shapiro–Wilk test, and variance homogeneity with Levene’s test. Two-group comparisons used Student’s *t*-test or Mann–Whitney test; multiple groups were compared with Kruskal–Wallis followed by Dwass-Steel-Critchlow-Fligner pairwise tests. Correlations were explored using Pearson or Spearman coefficients. A two-sided *p*-value < 0.05 was considered significant. Since no normative data are available for NEPs and PREPs elicited by 150 IDE, patient latencies were compared with those of age-matched controls; values exceeding the control mean by more than two standard deviations were classified as prolonged. NEP amplitudes were deemed abnormal only if unrecordable. Analyses were performed using Jamovi 2.3.28.

## 3 Results

### 3.1 Demographic and clinical characteristics of patients

Eight ATTRv-PN patients were included, with a median age of 78 years (range: 63–84) and a mean disease duration of 6.13 ± 2.23 years. Patients presented with varying severity of neuropathy, with an NIS ranging from 3 to 79.5 (Table 1).

**Table 1:** Demographic and clinical characteristics of patients with hereditary transthyretin amyloidosis with polyneuropathy.

Characteristic	ATTRv-PN (*N* = 8)
Age (years)	78 (range: 63–84; IQR: 74.25–80.25)
Males (*n*)	6
TTR variant (*n*)	p.Phe84Leu (3); p.Val50Met, late-onset (1); p.Ala140Thr (1); p.Tyr98Phe (1); p.Val142Ile (1); p.Ile88Leu (1)
ATTRv-PN therapy (*n*)	Patisiran (4); Inotersen (1); Tafamidis (3)
PND score (*n*)	I (5); II (1); IIIA (1); IIIB (1)
NIS (0–244)	28.13 ± 23.26
NIS-LL (0–88)	15.50 ± 14.26
CADT (% of the total score)	74.06 ± 15.35
COMPASS-31 (0–100)	20.50 ± 10.81
Norfolk-DN QoL (0–156)	38.00 ± 30.95
Neuropathic pain (*n*)	3

ATTRv-PN, hereditary transthyretin amyloidosis with polyneuropathy; TTR, transthyretin; PND, Polyneuropathy Disability score; NIS, Neuropathy Impairment Score; NIS-LL, NIS-lower limb; CADT, compound autonomic dysfunction test; COMPASS-31, composite autonomic symptom score-31; Norfolk-DN QoL, Norfolk Diabetic Neuropathy Quality of Life questionnaire.

Six asymptomatic carriers were included, with a median age of 50 years (range: 31–55), and a mean delta-PADO of −17.83 ± 11.87 years (Table 2). Only the two p.Arg144Cys carriers were less than 10 years younger than their PADO. None had an early-onset p.Val50Met variant.

**Table 2:** Demographic and clinical characteristics of asymptomatic carriers of TTR pathogenic variants.

Characteristic	Asymptomatic carriers (*N* = 6)
Age (years)	50 (range: 31–55; IQR: 46.5–53.5)
Males (*n*)	1
TTR variant (*n*)	p.Phe84Leu (4); p.Arg144Cys (2)
NIS (0–244)	0.50 ± 1.23
NIS-LL (0–88)	0.50 ± 1.23
CADT (% of the total score)	100 ± 0
COMPASS-31 (0–100)	0.67 ± 1.03

TTR, transthyretin; NIS, Neuropathy Impairment Score; NIS-LL, NIS-lower limb; CADT, compound autonomic dysfunction test; COMPASS-31, composite autonomic symptom score-31.

Fourteen healthy controls (seven males) participated in the study, with a median age of 67.5 years (range: 31–84; IQR: 52.5–79), matching the age distribution of patients and carriers.

All ATTRv patients and none of the presymptomatic carriers showed reduced CADT scores, while the COMPASS-31 scores ranged from 3 to 37 (median: 22) in symptomatic patients.

### 3.2 NEPs

NEP recordings in healthy subjects showed a series of components consistent with those previously described (8), with a mean N40 latency of 41.21 ± 3.13 ms and amplitude of 0.95 ± 0.54 μV.

All participants perceived a localized pinprick sensation from sensory threshold onward. Stimulation intensity was always kept below 5.00 mA, with a mean intensity of 1.88 ± 0.73 mA in controls, 2.15 ± 0.59 mA in ATTRv-PN patients, and 2.00 ± 1.11 mA in carriers. Longer delta-PADO in carriers correlated with lower perception threshold (*ρ* = −0.928, *p* = 0.008), suggesting that the threshold increases as disease onset approaches. Occasionally, perception diminished during stimulation, requiring a slight increase in intensity (<5.00 mA), typically when electrode-skin impedance dropped <10 kΩ. In such cases, stimulation was paused to cleanse the skin with ethanol and allow it to dry before repositioning the electrode. Stimulation with the 150 IDE caused a transient flare response.

**Figure 1:**
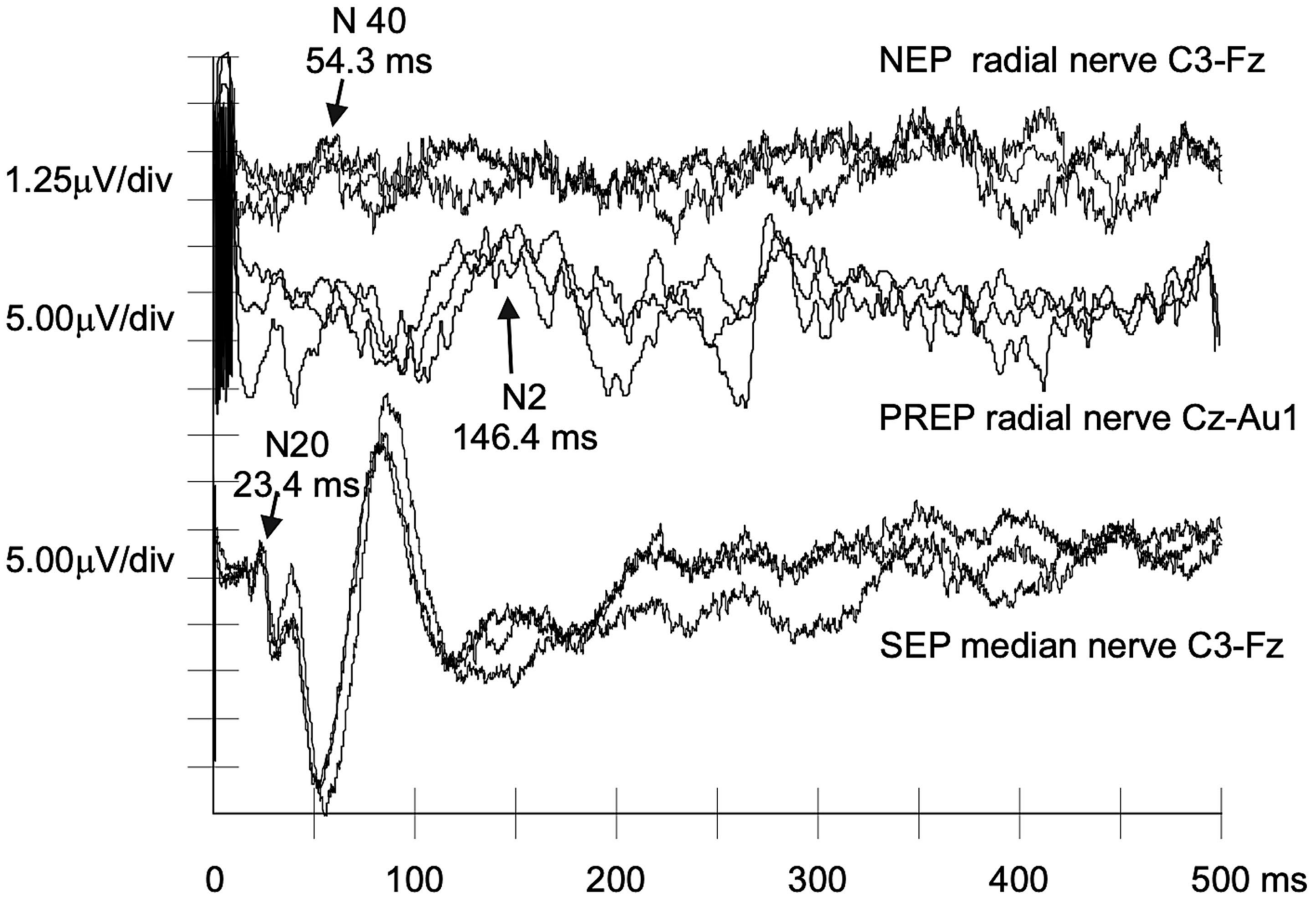
NEPs, PREPs, and SEPs recorded from a patient with late-onset p.Val50Met ATTRv-PN. This figure illustrates how NEPs may detect abnormalities in the nociceptive pathway in a symptomatic patient, while PREPs and SEPs show no changes. In this and other figures, negative deflections are depicted upwards. Recordings are from subject #P2. Each recording is represented by three traces, which are partial averages of approximately 150–200 responses each, superimposed to check the reproducibility of target components. From top to bottom, the first set of traces illustrates the NEPs obtained after stimulation of the dorsal skin of the right hand using the 150 IDE in a repetitive rhythmic mode. Component N40 is visible but considerably delayed and of low amplitude. The middle set shows the vertex PREPs after random stimulation, with a normal latency N2. The bottom set shows the traditional SEP after stimulation of the median nerve at the wrist, which is also within normal range.

**Figure 2:**
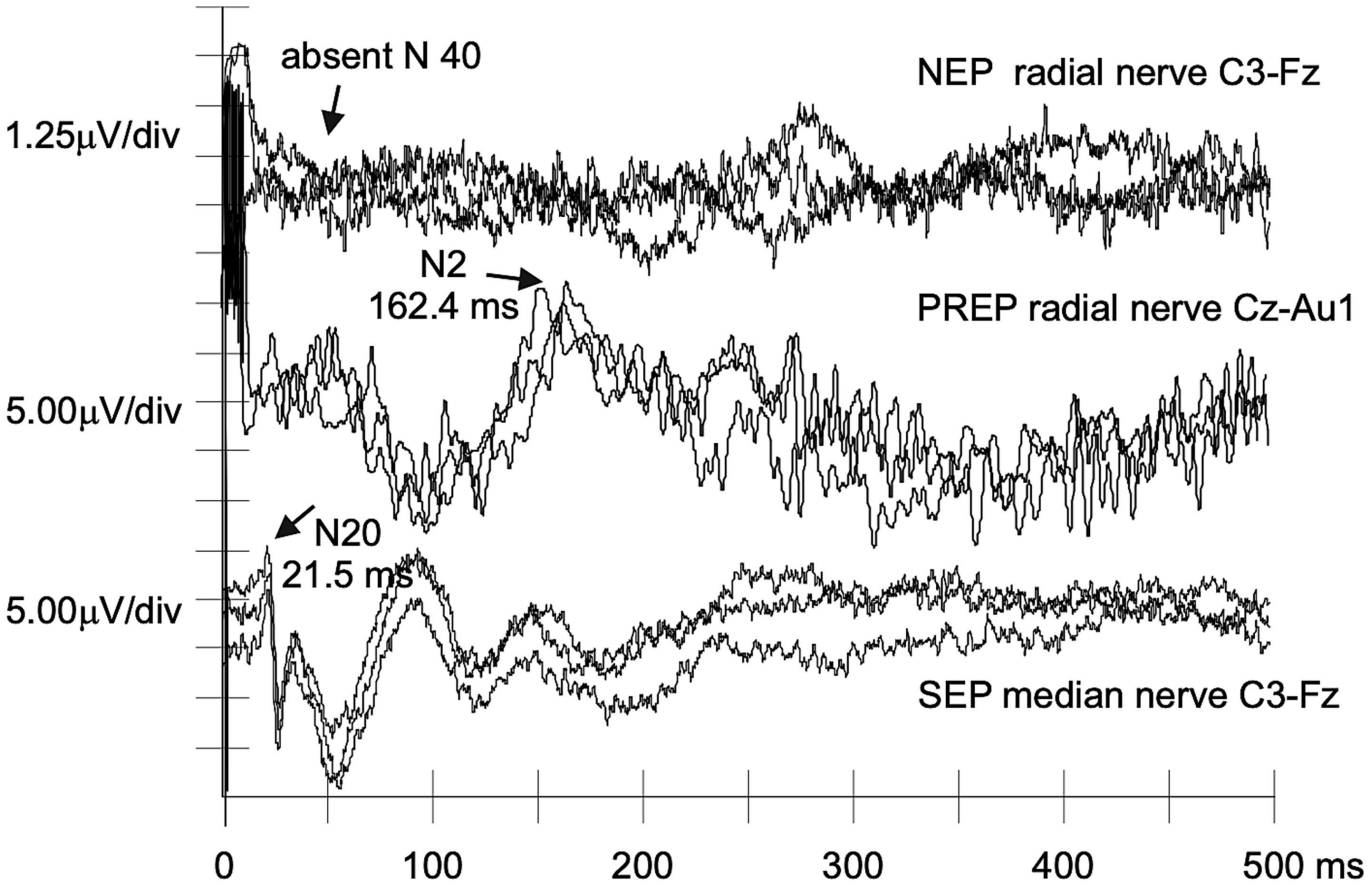
NEPs, PREPs, and SEPs recorded from a patient with p.Phe84Leu ATTRv-PN. A nonrecordable NEP N40 can be matched to still normal PREPs and SEPs in a case of clinically evident p.Phe84Leu ATTRv-PN (#P1).

Table 3 provides detailed ATTRv-PN findings. Five patients had absent NEPs, and one (#P2) exhibited a delayed N40 response (Figure 1). Traces from a patient with absent NEPs (#P1) are shown in Figure 2.

**Table 3:** Skin biopsy and neurophysiological findings in ATTRv-PN patients.

Patient Id	TTR variant	NIS (0–244)	Compass-31 (0–100)	Leg IENFD fibers/mm)	NEP latency (ms)	PREP latency (ms)		SEP latency (ms)		ESC
					N40	N1	N2	N20 radial 10CYC	N20 median 1CYC	
P1	p.Phe84Leu	22	22	6.8	**nr**	80.8	162.4	21.8	21.5	**red.**
P2	p.Val50Met	39.5	8	4.5	**54.3** ^**‡**^	92.4	146.4	**27.1**^**†**^	23.4	**red.**
P3	p.Phe84Leu	27	19	2.6	**nr**	**nr**	**nr**	23.1	**25.5** ^**†**^	**red.**
P4	p.Ala140Thr	79.5	37	–	**nr**	**nr**	**nr**	**31.5** ^**‡**^	**28.3** ^**‡**^	**red.**
P5	p.Phe84Leu	3	25	8.9	37.8	102.8	178.6	21.3	19.1	n
P6	p.Tyr98Phe	17	22	6.8	**nr**	84.6	198.2	**27.5** ^**†**^	23.5	**red.**
P7	p.Val142Ile	14	28	–	**nr**	132.2	**305.6** ^**†**^	**30.7** ^**‡**^	23.9	**red.**
P8	p.Ile88Leu	23	3	7.4	41.8	130.6	151.8	**27.1** ^**†**^	23.3	n

TTR, transthyretin; NIS, neuropathy impairment score; COMPASS-31, composite autonomic symptom score-31; IENFD, intraepidermal nerve fiber density; NEP, nociceptive evoked potential; PREP, pain-related evoked potential; SEP, somatosensory evoked potentials; ESC, electrochemical skin conductance.

n = normal; nr = not recordable; † = more than two standard deviations above the mean of healthy controls; ‡ = more than three standard deviations above the mean of healthy controls; red., reduced. For SEPs, the latencies of the N20 response are reported following stimulation of the radial nerve territory on the dorsum of the hand using trains of 10 pulses, as well as after median nerve stimulation at the wrist using single bursts.

Two patients (#P5 and #P8) with normal NEPs also had normal IENFD and ESC values. Both had mild neuropathy (PND I), with NIS scores of 3 (#P5) and 23 (#P8). One (#P8) carried the p.Ile88Leu TTR variant and exhibited predominant cardiac involvement. Patient #P7, who carried p.Val142Ile and had primarily cardiac involvement, showed absent NEPs and sudomotor dysfunction.

**Figure 3:**
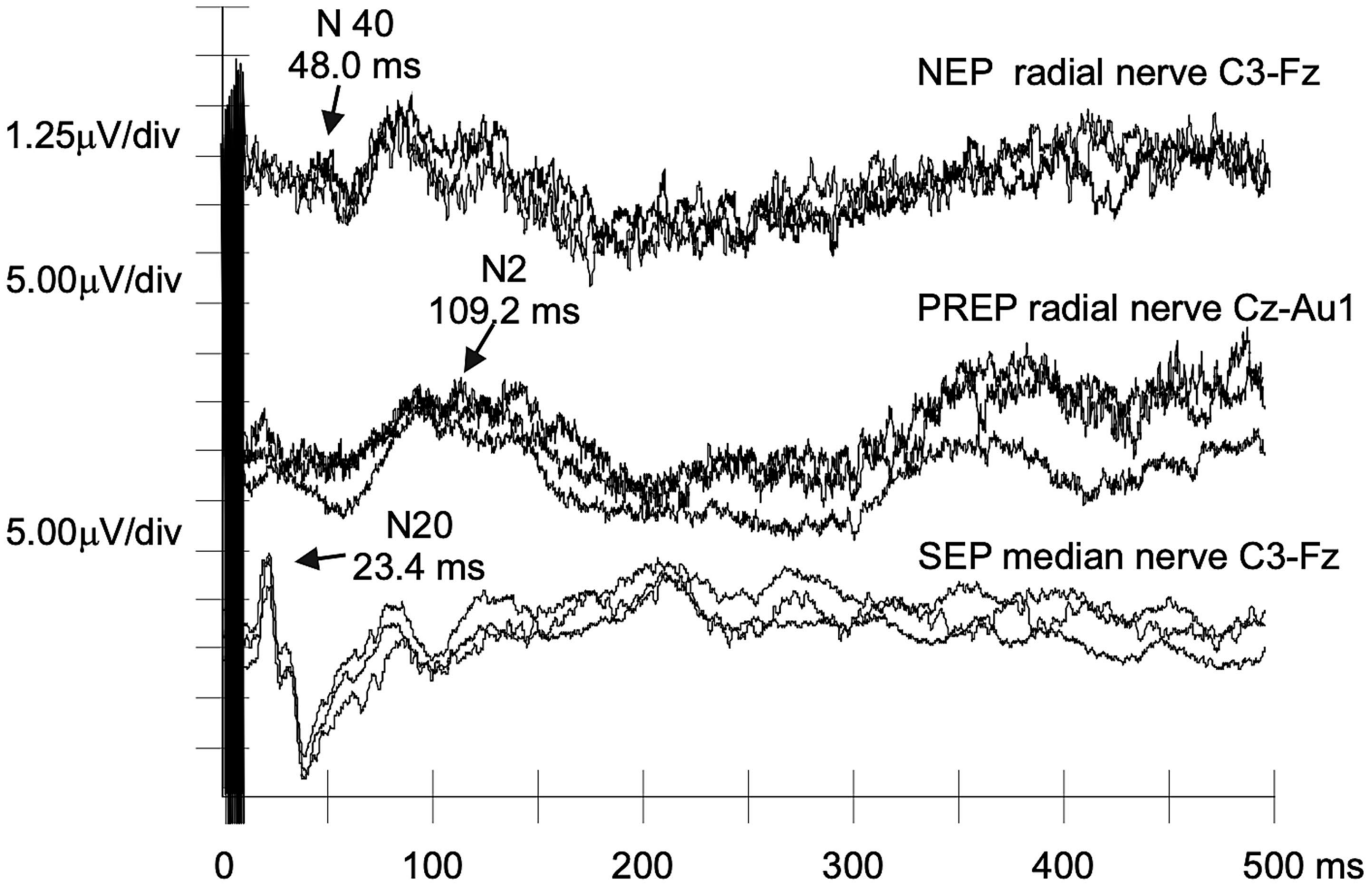
NEPs, PREPs, and SEPs recorded from an asymptomatic carrier of the p.Arg144Cys TTR mutation. Despite the absence of clinical symptoms, NEPs show increased N40 latency, with no abnormalities of PREPs and SEPs (case #A6). This further supports the potential sensitivity of NEPs to even limited damage to the nociceptive path.

Table 4 shows results for asymptomatic carriers. Two of six (#A2 and #A5, carrying p.Phe84Leu and p.Arg144Cys) lacked recognizable NEPs, and one (#A6, p.Arg144Cys) exhibited a delayed N40 response (Figure 3). These three had the shortest delta-PADO, suggesting proximity to disease onset.

**Table 4:** Skin biopsy and neurophysiological findings in asymptomatic carriers of TTR pathogenic variants.

Carrier Id	TTR variant	Delta-PADO (years)	Leg IENFD (fibers/mm)	NEP latencies (ms)	PREP latencies (ms)		SEP latencies (ms)		ESC
				N40	N1	N2	N20 radial 10CYC	N20 median 1CYC	
A1	p.Phe84Leu	−20	3.5	41.0	127.4	148.8	21.7	19.5	n
A2	p.Phe84Leu	−14	1.2	**nr**	150.2	245.0	22.7	20.2	n
A3	p.Phe84Leu	−22	–	40.0	104.3	153.2	20.3	19.4	n
A4	p.Phe84Leu	−38	–	38.4	105.2	141.0	21.9	19.8	n
A5	p.Arg144Cys	−5	–	**nr**	94	148.0	20.9	19.5	n
A6	p.Arg144Cys	−8	–	**48.0** ^**†**^	89.3	109.2	21.9	23.4	n

TTR, transthyretin; Delta-PADO, distance to predicted age of disease onset; IENFD, intraepidermal nerve fiber density; NEP, nociceptive evoked potential; PREP, pain-related evoked potential; SEP, somatosensory evoked potentials; ESC, electrochemical skin conductance.

n = normal; nr = not recordable; † = more than two standard deviations above the mean of healthy controls. For SEPs, the latencies of the N20 response are reported following stimulation of the radial nerve territory on the dorsum of the hand using trains of 10 pulses, as well as after median nerve stimulation at the wrist using single bursts.

### 3.3 PREPs

Random stimulation with 150 IDE elicited responses in all healthy subjects. N1 (C3/C4-Fz) showed a mean latency of 124.09 ± 31.77 ms and amplitude of 6.63 ± 4.12 μV, while N2 (Cz-Au) latency was 192.79 ± 53.93 ms with an amplitude of 6.04 ± 4.01 μV.

Two ATTRv-PN patients (#P3, #P4) had absent responses, and one (#P7) showed delayed N2; all had unrecordable NEPs and reduced ESC. Three others (#P1, #P2, #P6) had normal PREPs but unrecordable or delayed NEPs and low ESC (Table 3). No asymptomatic carriers showed abnormal PREPs (Table 4). Patients experiencing neuropathic pain had lower N1 amplitudes than those without (*t*(12) = 2.615, *p* = 0.023, *d* = 1.703).

### 3.4 SEPs

In healthy controls, radial SEPs showed N20 latency of 21.67 ± 2.19 ms and amplitude of 1.60 ± 2.54 μV.

Among ATTRv-PN patients, five (#P2, #P4, #P6, #P7, #P8) had prolonged radial N20 latencies, and two (#P3, #P4) had delayed median N20 latencies. All but one patient with delayed radial N20 also had absent or delayed NEPs; the exception (#P8, p.Ile88Leu) showed prolonged radial N20 but normal NEP N40 and median N20 latencies, had predominant cardiac involvement, and no evidence of small fiber impairment (Table 3). All asymptomatic carriers demonstrated normal nerve conduction studies and N20 latencies (Table 4).

### 3.5 ESC measurement

All healthy controls had normal Sudoscan results (Table 5). All but two ATTRv-PN patients (#P5, #P8) exhibited reduced ESC, whereas all asymptomatic carriers, including those with absent NEPs, had normal ESC.

**Table 5:** Electrochemical skin conductance values in healthy controls, patients with hereditary transthyretin amyloidosis, and asymptomatic carriers of TTR pathogenic variants.

Electrochemicalskin conductance	Healthy controls (*N* = 14)	ATTRv-PN (*N* = 8)	Asymptomatic carriers (*N* = 6)	Kruskall–Wallis (*p-*value)	*ε* ^2^
Right hand ESC (μS)	78.29 ± 5.11	40.75 ± 22.56	75.67 ± 8.91	**0.003**	0.421
Left hand ESC (μS)	79.21 ± 4.71	41.50 ± 21.79	76.50 ± 7.58	**0.002**	0.463
Right foot ESC (μS)	81.07 ± 5.30	36.75 ± 25.14	85.00 ± 5.02	**<0.001**	0.653
Left foot ESC (μS)	80.79 ± 5.95	40.13 ± 26.88	85.50 ± 3.89	**0.002**	0.449

ATTRv-PN, hereditary transthyretin amyloidosis with polyneuropathy; ESC, electrochemical skin conductance.

Pairwise comparisons using the Dwass-Steel-Critchlow-Fligner method revealed significantly lower ESC values in ATTRv-PN patients compared to healthy controls (right hand: *W* = 4.455, *p* = 0.005; left hand: *W* = 4.642, *p* = 0.003; right foot: *W* = 5.365, *p* < 0.001; left foot: *W* = 3.865, *p* = 0.017) and asymptomatic carriers (right hand: *W* = 3.651, *p* = 0.027; left hand: *W* = 3.747, *p* = 0.022; right foot: *W* = 4.391, *p* = 0.005; left foot: *W* = 4.122, *p* = 0.010). No significant differences were detected between asymptomatic carriers and healthy controls.

Patients with neuropathic pain had lower ESC than those without (right hand: *p* = 0.028; left hand: *p* = 0.016; right foot: *p* = 0.029; left foot: *p* = 0.024). Sudomotor dysfunction correlated with longer disease duration (right hand: *r* = −0.714, *p* = 0.004; left hand: *ρ* = −0.749, *p* = 0.002; right foot: *ρ* = −0.803, *p* < 0.001; left foot: *ρ* = −0.734, *p* = 0.003) and greater autonomic dysfunction as assessed with CADT (right hand: *ρ* = 0.703, *p* = 0.005; left hand: *ρ* = 0.715, *p* = 0.004; right foot: *ρ* = 0.708, *p* = 0.005; left foot: *ρ* = 0.730, *p* = 0.003).

## 4 Discussion

In recent years, the prognosis of ATTRv has improved remarkably with the introduction of therapies that slow the disease progression. Nevertheless, there is an unmet need for biomarkers capable of detecting disease onset and monitoring asymptomatic carriers. Small nerve fibers are typically the first to be affected, making neurophysiological evaluation of these fibers particularly valuable. The 150 IDE was developed to selectively stimulate intraepidermal nerve endings, enabling scalp recording of early NEPs, which reflect the first arrival of the afferent volley at the parietal sensory cortex. Unlike laser-evoked potentials (LEPs) and PREPs, which are event-related potentials influenced by attention and emotional-motivational pain processing, NEPs appear to provide a more direct measure of nociceptive function (8, 25). This pilot study explores NEPs as potential biomarkers of disease onset and progression in ATTRv.

### 4.1 NEPs as a marker of disease onset

Of the eight ATTRv-PN patients, five had absent N40 responses, and one showed a delayed N40. The two patients with normal NEPs had mild length-dependent polyneuropathy (PND score I) without evidence of small fiber involvement, as indicated by normal IENFD, PREPs, and ESC values. One carried the p.Phe84Leu variant, whereas the other carried the p.Ile88Leu variant and exhibited predominant cardiac involvement. In contrast, no N40 response was recorded from the patient carrying the p.Val142Ile variant, also typically associated with cardiac-predominant disease (26), who exhibited mild neuropathy affecting both large and small fibers, with an NIS of 14 and reduced ESC values. However, given the small number of patients and variants represented, these observations cannot be interpreted as evidence of variant-specific effects on NEPs. Among the six asymptomatic carriers, two had unrecordable N40 responses, and one showed a delayed response. NEP abnormalities were detected in carriers of the p.Phe84Leu and p.Arg144Cys variants, while none carried the early-onset p.Val50Met mutation, which is strongly associated with small fiber involvement (27). The three carriers with altered NEPs also had the shortest delta-PADO, with two falling below 10 years, a timeframe generally considered the window for early subclinical changes (15). Asymptomatic carriers with shorter delta-PADO also exhibited higher perception thresholds with the 150 IDE, possibly indicating that thresholds increase as clinical onset approaches.

Taken together, these findings suggest that NEPs may serve as early biomarkers of small fiber impairment in ATTRv, particularly in carriers approaching their PADO.

### 4.2 ESC changes as a marker of autonomic impairment and disease progression

Lefaucheur et al. (28) demonstrated that ESC values are reduced in most ATTRv patients and are decreased in 24% of asymptomatic carriers. In our study, none of the asymptomatic carriers exhibited decreased ESC, even those with unrecordable NEPs. However, we confirmed that ESC changes reflect the progression of neuropathy, as ESC values declined with longer disease duration. Lower CADT scores, reflecting more severe autonomic symptoms, correlated with reduced ESC values. These findings align with prior research showing foot ESC as an independent predictor of autonomic failure in p.Val50Met ATTRv (29). Luigetti et al. (30) also proposed ESC as a marker of disease progression in late-onset ATTRv patients, citing its inverse correlation with neuropathy severity and duration. In our study, ATTRv-PN patients with neuropathic pain had significantly lower ESC values than those without pain.

Our findings support the usefulness of Sudoscan for monitoring ATTRv patients. Although no ESC changes were observed in asymptomatic carriers, ESC decreased with longer disease duration and greater autonomic symptom severity.

### 4.3 Comprehensive neurophysiological results

ATTRv-PN patients had signs of impairment of Aδ (NEPs and PREPs), C (ESC), and Aβ (nerve conduction and SEPs) fibers. Only two patients, one with the p.Phe84Leu variant and the other with p.Ile88Leu, showed no small fiber involvement. In asymptomatic carriers, large fiber function was preserved (normal nerve conduction and SEPs), and ESC values were normal, despite NEP abnormalities (delayed or absent N40) observed in half of them. The decade preceding PADO is considered a critical window for monitoring, though in late-onset variants subclinical changes may appear even earlier (31). In this study, all asymptomatic carriers harbored late-onset variants and were generally young and far from their PADO. This likely contributed to the largely normal findings, whereas NEP abnormalities were observed in carriers closer to PADO.

Early small fiber involvement in ATTRv-PN is well established, particularly in early-onset p.Val50Met cases. Leonardi et al. (6) reported reduced distal IENFD in 50% of asymptomatic late-onset ATTRv carriers. Similarly, we found IENFD loss in asymptomatic carriers of the late-onset p.Phe84Leu mutation. As no prior studies have investigated NEPs in ATTRv, we also assessed PREPs, which also reflect Aδ fiber function. Our findings revealed lower PREP sensitivity, particularly during presymptomatic stages, as some individuals with altered NEPs and reduced ESC had normal PREPs, while all with abnormal PREPs also had altered NEPs. Regarding LEPs, which also assess Aδ fibers and share similar latencies with PREPs, Conceição et al. (32) found high specificity (97%) but low sensitivity (22%) in distinguishing controls from asymptomatic and mildly symptomatic p.Val50Met carriers. Another study reported abnormal LEPs in 35% of asymptomatic or minimally symptomatic carriers (28), lower than the 50% abnormal NEPs seen in our asymptomatic cohort. Several studies have examined the cutaneous silent period (CSP), an inhibitory reflex involving Aδ afferents and alpha motor neuron efferents. CSP latency was longer in ATTRv-PN patients versus controls and asymptomatic carriers, with no significant difference between the latter groups (33). In contrast, Luigetti et al. (34) observed a mild CSP latency difference between asymptomatic carriers and controls, but no difference in CSP duration.

Overall, NEPs, being independent of patient attention and highly reproducible, offer a valuable way to assess fast Aδ fibers in ATTRv-PN and asymptomatic carriers.

### 4.4 Main limitations and future directions

The main limitation of this study is the small sample size and participant heterogeneity. Nonetheless, relevant abnormalities were detected in ATTRv-PN patients and, to a lesser extent, in asymptomatic carriers. Expanding the participant pool and examining differences across TTR variants could strengthen the findings. Establishing normative values from a large healthy cohort is also essential. Another limitation is the lack of quantitative sensory testing, which was unavailable at the time of data collection but will be included in future assessments. As this is a cross-sectional study, a prospective longitudinal design is needed to confirm the reproducibility and progression of these findings over time. Assessing the timing of neurophysiological changes in asymptomatic carriers as they near their PADO would be of particular importance.

Given the challenges in assessing small fiber pathology and the genotypic and phenotypic heterogeneity of ATTRv, a multimodal approach remains essential for monitoring carriers and to differentiate truly asymptomatic carriers from those with subclinical denervation, asymptomatic amyloidosis, or overt amyloidosis (31), a distinction with major therapeutic implications. Skin biopsy plays a key role in detecting intraepidermal denervation and, in some cases, amyloid deposits (31, 35, 36). Denervation, however, appears many years before symptom onset and often without a clear correlation with clinical manifestations, likely reflecting cytotoxicity from prefibrillar TTR aggregates (31). The detection of amyloid deposits in the skin could serve as a biomarker of disease onset (35), although the sensitivity of Congo red staining remains uncertain. Corneal confocal microscopy and high-resolution MR neurography provide valuable morphological information on small fibers (37, 38); however, they do not directly assess function, and their clinical availability remains limited. Neurophysiological abnormalities in small fibers may precede Congo red–positive amyloid deposition, while conventional nerve conduction studies typically become abnormal only at advanced stages (31). Nevertheless, longitudinal changes in composite sensory neurophysiological scores may anticipate phenoconversion by up to 2 years (39). Combining complementary techniques to evaluate small myelinated Aδ fibers, such as NEPs or PREPs, and unmyelinated C fibers, such as ESC, may be particularly useful for follow-up, as these methods are relatively simple and feasible in clinical practice. Neurofilament light chain (NfL) is also emerging as a promising biomarker in ATTRv-PN (40, 41), and its combination with small fiber assessments may improve monitoring of asymptomatic carriers.

### 4.5 Conclusion

In this study, the 150 IDE was employed to assess small fiber function in ATTRv. NEPs elicited by this electrode appear to be a promising tool for evaluating small fiber involvement, even during presymptomatic stages of ATTRv. Our findings suggest that NEPs may serve as an early biomarker of disease onset, as demonstrated by alterations in asymptomatic carriers nearing clinical manifestations. Clinical evaluations combined with NEPs, ESC, and skin biopsy provide complementary perspectives on small fiber pathology in ATTRv. Since the 150 IDE enables a cost-effective evaluation of small fibers using standard electromyographic equipment, this integrated approach could be extended to other neurological disorders.

## Data Availability

The raw data supporting the conclusions of this article will be made available by the authors, without undue reservation.

## Glossary

150 IDE: interdigitated electrode with rail gaps of 150 μm
ATTRv: hereditary transthyretin amyloidosis
ATTRv-PN: hereditary transthyretin amyloidosis with polyneuropathy
CADT: compound autonomic dysfunction test
COMPASS-31: composite autonomic symptom score-31
CSP: cutaneous silent period
DN4: Douleur Neuropathique 4 Scale
ESC: electrochemical skin conductance
IENFD: intraepidermal nerve fiber density
LEPs: laser-evoked potentials
NEPs: nociceptive evoked potentials
NIS: neuropathy impairment score
NIS-LL: neuropathy impairment score—lower limb
Norfolk-DN QoL: Norfolk Diabetic Neuropathy Quality of Life Questionnaire
PADO: predicted age of symptomatic disease onset
PND: polyneuropathy disability score
PREPs: pain-related evoked potentials
QST: quantitative sensory testing
SEPs: somatosensory evoked potentials
TTR: transthyretin

## References

[1] Gertz MA. Hereditary ATTR amyloidosis: burden of illness and diagnostic challenges. Am J Manag Care. (2017) 23:S107–12. PMID: 28978215

[2] Tozza S Severi D Spina E Iovino A Aruta F Ruggiero L . The neuropathy in hereditary transthyretin amyloidosis: a narrative review. J Peripher Nerv Syst. (2021) 26:155–9. doi: 10.1111/jns.12451, PMID: 33960565 PMC8360044

[3] Planté-Bordeneuve V Said G. Familial amyloid polyneuropathy. Lancet Neurol. (2011) 10:1086–97. doi: 10.1016/S1474-4422(11)70246-0, PMID: 22094129

[4] Adams D Suhr OB Hund E Obici L Tournev I Campistol JM . First European consensus for diagnosis, management, and treatment of transthyretin familial amyloid polyneuropathy. Curr Opin Neurol. (2016) 29:S14–26. doi: 10.1097/WCO.0000000000000289, PMID: 26734952 PMC4739312

[5] Adams D Ando Y Beirão JM Coelho T Gertz MA Gillmore JD . Expert consensus recommendations to improve diagnosis of ATTR amyloidosis with polyneuropathy. J Neurol. (2021) 268:2109–22. doi: 10.1007/s00415-019-09688-0, PMID: 31907599 PMC8179912

[6] Leonardi L Costanzo R Forcina F Morino S Antonini G Salvetti M . Quantitative sensory testing and skin biopsy findings in late-onset ATTRv presymptomatic carriers: relationships with predicted time of disease onset (PADO). J Peripher Nerv Syst. (2023) 28:390–7. doi: 10.1111/jns.12586, PMID: 37535421

[7] Leandri M Marinelli L Siri A Pellegrino L. Micropatterned surface electrode for massive selective stimulation of intraepidermal nociceptive fibres. J Neurosci Methods. (2018) 293:17–26. doi: 10.1016/J.JNEUMETH.2017.08.032, PMID: 28899650

[8] Leandri M Di Stefano G Truini A Marinelli L. Early nociceptive evoked potentials (NEPs) recorded from the scalp. Clin Neurophysiol. (2021) 132:2896–906. doi: 10.1016/J.CLINPH.2021.05.027, PMID: 34226125

[9] Di Stefano G Di Lionardo A La Cesa S Di Pietro G Fasolino A Galosi E . The new micropatterned interdigitated electrode for selective assessment of the nociceptive system. Eur J Pain. (2020) 24:956–66. doi: 10.1002/EJP.1545, PMID: 32064700

[10] Katsarava Z Ayzenberg I Sack F Limmroth V Diener HC Kaube H. A novel method of eliciting pain-related potentials by transcutaneous electrical stimulation. Headache. (2006) 46:1511–7. doi: 10.1111/J.1526-4610.2006.00446.X, PMID: 17115984

[11] Inui K Kakigi R. Pain perception in humans: use of intraepidermal electrical stimulation. J Neurol Neurosurg Psychiatry. (2012) 83:551–6. doi: 10.1136/JNNP-2011-301484, PMID: 22138180

[12] Inui K Tran TD Hoshiyama M Kakigi R. Preferential stimulation of Adelta fibers by intra-epidermal needle electrode in humans. Pain. (2002) 96:247–52. doi: 10.1016/S0304-3959(01)00453-5, PMID: 11972996

[13] Perchet C Frot M Charmarty A Flores C Mazza S Magnin M . Do we activate specifically somatosensory thin fibres with the concentric planar electrode? A scalp and intracranial EEG study. Pain. (2012) 153:1244–52. doi: 10.1016/J.PAIN.2012.03.004, PMID: 22497800

[14] Lauria G. Small fibre neuropathies. Curr Opin Neurol. (2005) 18:591–7. doi: 10.1097/01.WCO.0000177330.35147.70, PMID: 16155446

[15] Conceição I Damy T Romero M Galán L Attarian S Luigetti M . Early diagnosis of ATTR amyloidosis through targeted follow-up of identified carriers of *TTR* gene mutations*. Amyloid. (2019) 26:3–9. doi: 10.1080/13506129.2018.1556156, PMID: 30793974

[16] Dyck PJB González-Duarte A Obici L Polydefkis M Wiesman JF Antonino I . Development of measures of polyneuropathy impairment in hATTR amyloidosis: from NIS to mNIS + 7. J Neurol Sci. (2019) 405:116424. doi: 10.1016/J.JNS.2019.116424, PMID: 31445300

[17] Berk JL Suhr OB Obici L Sekijima Y Zeldenrust SR Yamashita T . Repurposing diflunisal for familial amyloid polyneuropathy: a randomized clinical trial. JAMA. (2013) 310:2658–67. doi: 10.1001/jama.2013.283815, PMID: 24368466 PMC4139164

[18] Denier C Ducot B Husson H Lozeron P Adams D Meyer L . A brief compound test for assessment of autonomic and sensory-motor dysfunction in familial amyloid polyneuropathy. J Neurol. (2007) 254:1684–8. doi: 10.1007/s00415-007-0617-5, PMID: 18074076

[19] Treister R O’Neil K Downs HM Oaklander AL. Validation of the composite autonomic symptom scale 31 (COMPASS-31) in patients with and without small fiber polyneuropathy. Eur J Neurol. (2015) 22:1124–30. doi: 10.1111/ENE.12717 25907824 PMC4464987

[20] Vinik EJ Hayes RP Oglesby A Bastyr E Barlow P Ford-Molvik SL . The development and validation of the Norfolk QOL-DN, a new measure of patients’ perception of the effects of diabetes and diabetic neuropathy. Diabetes Technol Ther. (2005) 7:497–508. doi: 10.1089/dia.2005.7.497, PMID: 15929681

[21] Bouhassira D Attal N Alchaar H Boureau F Brochet B Bruxelle J . Comparison of pain syndromes associated with nervous or somatic lesions and development of a new neuropathic pain diagnostic questionnaire (DN4). Pain. (2005) 114:29–36. doi: 10.1016/J.PAIN.2004.12.010, PMID: 15733628

[22] Mayaudon H Miloche PO Bauduceau B. Une nouvelle méthode simple pour évaluer la function sudomotrice intérêt dans le diabète de type 2. Diabetes Metab. (2010) 36:450–4. doi: 10.1016/j.diabet.2010.05.004 20739207

[23] Lauria G Hsieh ST Johansson O Kennedy WR Leger JM Mellgren SI . European Federation of Neurological Societies/peripheral nerve society guideline on the use of skin biopsy in the diagnosis of small fiber neuropathy. Report of a joint task force of the European Federation of Neurological Societies and the peripheral nerve society. J Peripher Nerv Syst. (2010) 15:79–92. doi: 10.1111/J.1529-8027.2010.00269.X 20626771

[24] Bakkers M Merkies ISJ Lauria G Devigili G Penza P Lombardi R . Intraepidermal nerve fiber density and its application in sarcoidosis. Neurology. (2009) 73:1142–8. doi: 10.1212/WNL.0B013E3181BACF05, PMID: 19805731

[25] Blake DT. Encephalographic studies of central nociceptive activation just got a bit easier. Clin Neurophysiol. (2021) 132:2890–1. doi: 10.1016/j.clinph.2021.08.007, PMID: 34583884

[26] Gentile L Di Bella G Minutoli F Cucinotta F Obici L Mussinelli R . Description of a large cohort of Caucasian patients with V122I ATTRv amyloidosis: neurological and cardiological features. J Peripher Nerv Syst. (2020) 25:273–8. doi: 10.1111/jns.12385, PMID: 32395865

[27] Manganelli F Fabrizi GM Luigetti M Mandich P Mazzeo A Pareyson D. Hereditary transthyretin amyloidosis overview. Neurol Sci. (2022) 43:595–604. doi: 10.1007/S10072-020-04889-2, PMID: 33188616 PMC9780126

[28] Lefaucheur J-P Ng Wing Tin S Kerschen P Damy T Planté-Bordeneuve V. Neurophysiological markers of small fibre neuropathy in TTR-FAP mutation carriers. J Neurol. (2013) 260:1497–503. doi: 10.1007/s00415-012-6816-8, PMID: 23306657

[29] Castro J Miranda B Castro I de Carvalho M Conceição I. The diagnostic accuracy of Sudoscan in transthyretin familial amyloid polyneuropathy. Clin Neurophysiol. (2016) 127:2222–7. doi: 10.1016/J.CLINPH.2016.02.013, PMID: 27072093

[30] Luigetti M Bisogni G Romano A Di Paolantonio A Barbato F Primicerio G . Sudoscan in the evaluation and follow-up of patients and carriers with TTR mutations: experience from an Italian Centre. Amyloid. (2018) 25:242–6. doi: 10.1080/13506129.2018.1545640, PMID: 30638075

[31] Beauvais D Labeyrie C Cauquil C Francou B Eliahou L Not A . Detailed clinical, physiological and pathological phenotyping can impact access to disease-modifying treatments in ATTR carriers. J Neurol Neurosurg Psychiatry. (2024) 95:489–99. doi: 10.1136/jnnp-2023-332180, PMID: 37875336 PMC11103288

[32] Conceição I Costa J Castro J de Carvalho M. Neurophysiological techniques to detect early small-fiber dysfunction in transthyretin amyloid polyneuropathy. Muscle Nerve. (2014) 49:181–6. doi: 10.1002/MUS.23901, PMID: 23681916

[33] Cambieri C Libonati L Moret F Tartaglia G Garibaldi M Chimenti C . The silent period for small fiber sensory neuropathy assessment in a mixed cohort of transthyretin-mediated amyloidosis. Biomedicine. (2022) 10. doi: 10.3390/biomedicines10092073, PMID: 36140174 PMC9495326

[34] Luigetti M Di Paolantonio A Guglielmino V Romano A. Cutaneous silent period in ATTRv carriers: a possible early marker of nerve damage? Neurol Sci. (2022) 43:6979–82. doi: 10.1007/S10072-022-06317-Z, PMID: 35943637

[35] Leonardi L Adam C Beaudonnet G Beauvais D Cauquil C Not A . Skin amyloid deposits and nerve fiber loss as markers of neuropathy onset and progression in hereditary transthyretin amyloidosis. Eur J Neurol. (2022) 29:1477–87. doi: 10.1111/ENE.15268, PMID: 35100482

[36] Freeman R Gonzalez-Duarte A Barroso F Campagnolo M Rajan S Garcia J . Cutaneous amyloid is a biomarker in early ATTRv neuropathy and progresses across disease stages. Ann Clin Transl Neurol. (2022) 9:1370–83. doi: 10.1002/ACN3.51636, PMID: 35945901 PMC9463946

[37] Kollmer J Sahm F Hegenbart U Purrucker JC Kimmich C Schönland SO . Sural nerve injury in familial amyloid polyneuropathy. Neurology. (2017) 89:475–84. doi: 10.1212/WNL.0000000000004178, PMID: 28679600

[38] Thimm A Carpinteiro A Oubari S Papathanasiou M Kessler L Rischpler C . Corneal confocal microscopy identifies corneal nerve loss and increased Langerhans cells in presymptomatic carriers and patients with hereditary transthyretin amyloidosis. J Neurol. (2023) 270:3483–91. doi: 10.1007/s00415-023-11689-z, PMID: 37014422 PMC10267010

[39] Castro J Miranda B de Castro I Conceição I. Changes in nerve conduction studies predate clinical symptoms onset in early onset Val30Met hereditary ATTR amyloidosis. Eur J Neurol. (2022) 29:826–32. doi: 10.1111/ene.15176, PMID: 34751997

[40] Carroll AS Razvi Y O’Donnell L Veleva E Heslegrave A Zetterberg H . Serum neurofilament light chain in hereditary transthyretin amyloidosis: validation in real-life practice. Amyloid. (2024) 31:95–104. doi: 10.1080/13506129.2024.2313218, PMID: 38348665

[41] Galosi E Costanzo R Forcina F Morino S Antonini G Salvetti M . Serum neurofilament light chain levels correlate with small fiber related parameters in patients with hereditary transthyretin amyloidosis with polyneuropathy (ATTRv-PN). Neurol Sci. (2024) 45:5023–32. doi: 10.1007/s10072-024-07562-0, PMID: 38700599 PMC11422273

